# Enzalutamide versus bicalutamide in patients with nonmetastatic castration-resistant prostate cancer: a prespecified subgroup analysis of the STRIVE trial

**DOI:** 10.1038/s41391-021-00465-7

**Published:** 2021-10-07

**Authors:** David F. Penson, Andrew J. Armstrong, Raoul S. Concepcion, Neeraj Agarwal, Carl A. Olsson, Lawrence I. Karsh, Curtis J. Dunshee, William Duggan, Qi Shen, Jennifer Sugg, Gabriel P. Haas, Celestia S. Higano

**Affiliations:** 1 Department of Urology, Vanderbilt University Medical Center, Nashville, TN USA; 2 Division of Medical Oncology, Department of Medicine, Duke Cancer Institute Center for Prostate and Urologic Cancers, Duke University, Durham, NC USA; 3 The Comprehensive Prostate Center, Nashville, TN USA; 4 Huntsman Cancer Institute, University of Utah, Salt Lake City, UT USA; 5 Accumed Research Associates, Garden City, NY USA; 6 The Urology Center of Colorado, Denver, CO USA; 7 Urological Associates of Southern Arizona, Tucson, AZ USA; 8 Global Product Development, Pfizer Inc., Groton, CT USA; 9 Global Product Development, Pfizer Inc., Collegeville, PA USA; 10 Biostatistics, Astellas Pharma, Inc., Northbrook, IL USA; 11 Global Development, Astellas Pharma, Inc., Northbrook, IL USA; 12 Department of Medicine, Division of Oncology, University of Washington and Fred Hutchinson Cancer Research Center, Seattle, WA USA

**Keywords:** Cancer therapy, Cancer therapy, Prostate cancer, Outcomes research, Prostate cancer

## Abstract

**Background:**

In the phase 2, randomized, double-blind STRIVE trial, enzalutamide significantly reduced the risk of prostate cancer progression or death versus bicalutamide in patients with metastatic castration-resistant prostate cancer (mCRPC) and nonmetastatic CRPC (nmCRPC). The objective of this protocol-specified subgroup analysis of STRIVE was to investigate the benefit of enzalutamide versus bicalutamide specifically in patients with nmCRPC.

**Methods:**

Patients (*N* = 139) were stratified by disease stage and randomized to enzalutamide 160 mg/day plus androgen deprivation therapy (ADT; *n* = 70) or bicalutamide 50 mg/day plus ADT (*n* = 69).

**Results:**

Baseline characteristics of patients with nmCRPC were comparable between groups. At a median of 17 months follow-up, enzalutamide reduced the risk of progression or death by 76% versus bicalutamide in patients with nmCRPC (hazard ratio [HR], 0.24; 95% CI 0.14–0.42). Enzalutamide reduced risk of prostate-specific antigen progression by 82% versus bicalutamide in patients with nmCRPC (HR, 0.18; 95% CI 0.10–0.34). The most frequently reported adverse events by patients receiving enzalutamide were fatigue (36.2%), hot flush (20.3%), decreased appetite (17.4%), dizziness (17.4%), and nausea (17.4%).

**Conclusions:**

This STRIVE subgroup analysis of patients with nmCRPC illustrates the benefit of enzalutamide in reducing the risk of progression or death versus bicalutamide in patients with nmCRPC.

**Trial registration:**

ClinicalTrials.gov identifier NCT01664923.

## Introduction

In the phase 2, randomized, double-blind STRIVE trial, enzalutamide significantly reduced the risk of prostate cancer progression or death versus bicalutamide, in patients with metastatic castration-resistant prostate cancer (mCRPC) and nonmetastatic CRPC (nmCRPC) [1]. Several phase 3 trials have evaluated the efficacy of novel hormonal therapies (NHTs) in patients with nmCRPC, including enzalutamide (PROSPER) [2, 3], apalutamide (SPARTAN) [4–6], and darolutamide (ARAMIS) [7, 8]. Despite evidence of the efficacy of enzalutamide worldwide, patients with nmCRPC are still commonly treated with bicalutamide [9]. The objective of this prespecified subgroup analysis of STRIVE was to investigate the clinical benefit of enzalutamide versus bicalutamide in patients with nmCRPC by reporting progression-free survival (PFS), time to prostate-specific antigen progression (TTPP), and associated safety data not previously reported in STRIVE.

## Methods

The STRIVE trial, described previously [1], was a randomized, double-blind, phase 2 study of enzalutamide 160 mg/day plus androgen deprivation therapy (ADT; *n* = 70) versus bicalutamide 50 mg/day plus ADT (*n* = 69) in patients with nmCRPC or mCRPC. The primary endpoint of this subgroup analysis was PFS, defined as time from randomization to prostate-specific antigen (PSA) progression, or death due to any cause. Secondary endpoints included TTPP. Radiographic PFS (rPFS), defined as time from randomization to the earliest evidence of radiographic progression or death on study, was also evaluated as an endpoint. Kaplan-Meier curves and medians were calculated for these endpoints, and HRs were estimated by using a Cox regression model. A two-sided Cochran-Mantel-Haenszel test was used to compare PSA response rates for enzalutamide and bicalutamide. Safety data were evaluated for the nmCRPC subset and not adjusted for time on the study drug.

## Results

Baseline patient characteristics in the nmCRPC subgroup were comparable between cohorts (enzalutamide *n* = 70, bicalutamide *n* = 69), except patients in the bicalutamide group were older (median age, 77.0 years vs 73.5 years) and had longer PSA doubling times (PSADT; median, 5.3 months vs 3.9 months) (Supplementary Table S1). Consistent with the overall STRIVE analysis, the median time on treatment in this subpopulation was longer for patients receiving enzalutamide versus those receiving bicalutamide (17.8 months vs 12.3 months).

**Fig. 1:**
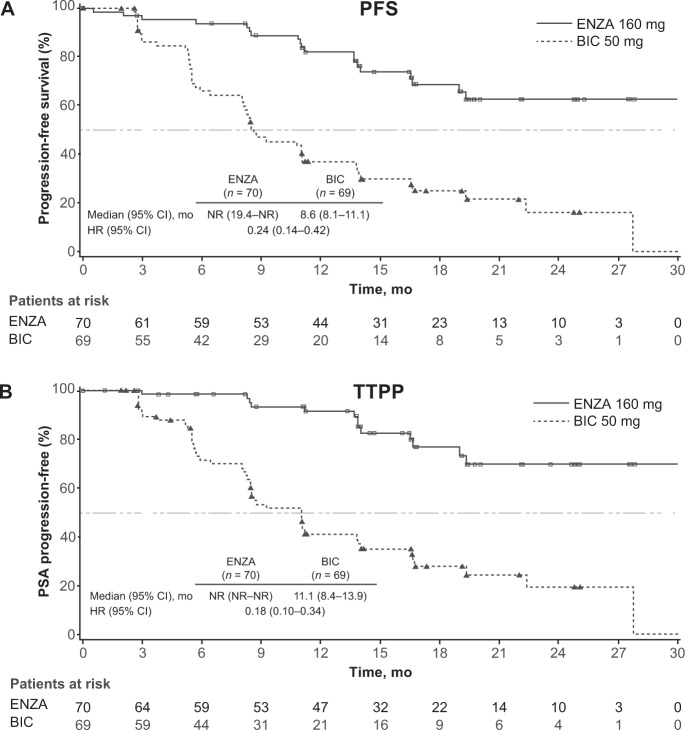
Progression Outcomes in STRIVE study. **A** Progression-free survival (PFS) and **B** time to PSA progression (TTPP) in the STRIVE nonmetastatic castration-resistant prostate cancer (nmCRPC) subpopulation. P-value was calculated using an unstratified log-rank test; Hazard ratio was calculated using a unstratified Cox regression model with treatment as the covariate and is relative to bicalutamide with <1 favoring enzalutamide. *BIC* bicalutamide; *ENZA* enzalutamide; *HR* hazard ratio; *NR* not reached; *mo* months.

At a median follow-up of 17 months, enzalutamide significantly reduced the risk of progression or death by 76% compared with bicalutamide in patients with nmCRPC (hazard ratio [HR], 0.24; 95% CI 0.14–0.42; *P* < 0.0001; Fig. 1A). Enzalutamide reduced the risk of PSA progression by 82% versus bicalutamide in patients with nmCRPC (HR, 0.18; 95% CI 0.10–0.34; *P* < 0.0001; Fig. 1B).

The benefit from enzalutamide on PFS was consistent across all subgroups, including age, Eastern Cooperative Oncology Group performance status, Gleason score, baseline PSA level, baseline hemoglobin level, use of bone-targeting therapy, and presence of nodal disease at study entry (Supplementary Fig. S1). PSADT subgroups ≥6–12 months did not reach statistical significance for PFS (Supplementary Fig. S1). Similarly, baseline PSADT subgroups <10 months and ≥10 months did not reach statistical significance (Supplementary Fig. S2). The benefit of enzalutamide on rPFS as an endpoint was consistent across all subgroups (Supplementary Fig. 3).

The benefit of enzalutamide on time to PSA progression was consistent across patients with PSADT <10 months and ≥10 months at baseline. Enzalutamide-treated patients with PSADT of <10 months report less PSA progression compared with those treated with bicalutamide (19.4% vs 76.0%, HR, 0.156; 95% CI 0.080–0.301; *P* < 0.0001; Supplementary Table S2).

Enzalutamide-treated patients in both PSADT subgroups reported higher PSA response rates of ≥50% decrease from baseline (<10 months, 89.7% response; ≥10 months, 100% response rate) compared with bicalutamide groups (<10 months, 38.0% response rate; ≥10 months, 66.7% response rate; Supplementary Table S3).

The most frequent adverse events (AEs; unadjusted for treatment exposure) in patients receiving enzalutamide versus bicalutamide were fatigue (36.2% vs 21.7%), hot flush (20.3% vs 2.9%), decreased appetite (17.4% vs 5.8%), dizziness (17.4% vs 4.3%), and nausea (17.4% vs 13.0%). More frequently reported AEs with bicalutamide versus enzalutamide were constipation (17.4% vs 7.2%), urinary tract infection (15.9% vs 1.4%), and diarrhea (11.6% vs 8.7%). The most frequent grade ≥3 AEs in the enzalutamide group versus bicalutamide were fatigue (5.8% vs 2.9%), arthralgia (4.3% vs 1.4%), congestive cardiac failure (4.3% vs 1.4%), and hypertension (4.3% vs 2.9%). The most frequent grade ≥3 AEs in the bicalutamide group versus enzalutamide were syncope (4.3% vs 2.9%) and urinary retention (4.3% vs 0%).

Two deaths were reported in the enzalutamide group: a 92-year-old patient died of cardiopulmonary arrest, assessed as probably related to study drug by the investigator, and an 87-year-old patient died of general health deterioration considered unrelated to study drug.

## Discussion

The STRIVE trial reported enzalutamide improved both median PFS (19.4 months vs 5.7 months; *P* < 0.0001) and median TTPP (estimate not reached vs 8.3 months; *P* < 0.0001) versus bicalutamide, consistently in patients with nmCRPC and mCRPC [1]. It should be noted that overall survival was not an endpoint of the STRIVE trial.

The benefit of enzalutamide in this population is consistent with the efficacy of enzalutamide versus placebo in the PROSPER trial of patients with nmCRPC [2, 3] in which OS was a secondary endpoint. The safety profile of enzalutamide in nmCRPC is consistent with the overall STRIVE population [2, 3, 10]. One patient with underlying hypertension died in the enzalutamide group of cardiopulmonary arrest. The subgroup analysis reveals a slight elevation in the incidence of hypertension in the enzalutamide group versus bicalutamide (11.6% vs 7.2%, unadjusted for treatment exposure). These findings call for close management of enzalutamide-treated patients predisposed to hypertension or cardiovascular disease. Overall, these findings are particularly informative for clinicians who continue to prescribe bicalutamide in patients with nmCRPC.

In conclusion, this STRIVE subgroup analysis is the only prospectively conducted study of patients with nmCRPC comparing an NHT to bicalutamide. In addition, enzalutamide is the only NHT that has shown improved efficacy compared with bicalutamide for metastatic hormone-sensitive prostate cancer [10], nmCRPC [1], and mCRPC [1].

### Data Sharing Statement

Upon request and subject to certain criteria, conditions, and exceptions (see https://www.pfizer.com/science/clinical-trials/trial-data-and-results for more information), Pfizer will provide access to individual de-identified participant data from Pfizer-sponsored global interventional clinical studies conducted for medicines, vaccines, and medical devices (1) for indications that have been approved in the US and/or EU or (2) in programs that have been terminated (i.e., development for all indications has been discontinued). Pfizer will also consider requests for the protocol, data dictionary, and statistical analysis plan. Data may be requested from Pfizer trials 24 months after study completion. The de-identified participant data will be made available to researchers whose proposals meet the research criteria and other conditions, and for which an exception does not apply, via a secure portal. To gain access, data requestors must enter into a data access agreement with Pfizer.

## Supplementary information

Supplemental Materials
