# Correction: Enzalutamide versus bicalutamide in patients with nonmetastatic castration-resistant prostate cancer: a prespecified subgroup analysis of the STRIVE trial

**DOI:** 10.1038/s41391-021-00477-3

**Published:** 2021-11-30

**Authors:** David F. Penson, Andrew J. Armstrong, Raoul S. Concepcion, Neeraj Agarwal, Carl A. Olsson, Lawrence I. Karsh, Curtis J. Dunshee, William Duggan, Qi Shen, Jennifer Sugg, Gabriel P. Haas, Celestia S. Higano

**Affiliations:** 1 Department of Urology, Vanderbilt University Medical Center, Nashville, TN USA; 2 Division of Medical Oncology, Department of Medicine, Duke Cancer Institute Center for Prostate and Urologic Cancers, Duke University, Durham, NC USA; 3 The Comprehensive Prostate Center, Nashville, TN USA; 4 Huntsman Cancer Institute, University of Utah, Salt Lake City, UT USA; 5 Accumed Research Associates, Garden City, NY USA; 6 The Urology Center of Colorado, Denver, CO USA; 7 Urological Associates of Southern Arizona, Tucson, AZ USA; 8 Global Product Development, Pfizer Inc., Groton, CT USA; 9 Global Product Development, Pfizer Inc., Collegeville, PA USA; 10 Biostatistics, Astellas Pharma, Inc., Northbrook, IL USA; 11 Global Development, Astellas Pharma, Inc., Northbrook, IL USA; 12 Department of Medicine, Division of Oncology, University of Washington and Fred Hutchinson Cancer Research Center, Seattle, WA USA

**Keywords:** Cancer therapy, Cancer therapy, Prostate cancer, Outcomes research, Prostate cancer

Correction to: *Prostate Cancer and Prostatic Diseases* 10.1038/s41391-021-00465-7, published online 7 October 2021

The article Enzalutamide versus bicalutamide in patients with nonmetastatic castration-resistant prostate cancer: a prespecified subgroup analysis of the STRIVE trial, written by David F. Penson, Andrew J. Armstrong, Raoul S. Concepcion, Neeraj Agarwal, Carl A. Olsson, Lawrence I. Karsh, Curtis J. Dunshee, William Duggan, Qi Shen, Jennifer Sugg, Gabriel P. Haas and Celestia S. Higano, was originally published electronically on the publisher’s internet portal on 07 October 2021 without open access. With the author(s)’ decision to opt for Open Choice the copyright of the article changed on 20 October 2021 to © The Authors 2021 and the article is forthwith distributed under a Creative Commons Attribution 4.0 International License, which permits use, sharing, adaptation, distribution and reproduction in any medium or format, as long as you give appropriate credit to the original author(s) and the source, provide a link to the Creative Commons licence, and indicate if changes were made. The images or other third party material in this article are included in the article’s Creative Commons licence, unless indicated otherwise in a credit line to the material. If material is not included in the article’s Creative Commons licence and your intended use is not permitted by statutory regulation or exceeds the permitted use, you will need to obtain permission directly from the copyright holder. To view a copy of this licence, visit http://creativecommons.org/licenses/by/4.0.

