# Supplementary material for: Enzalutamide versus bicalutamide in patients with nonmetastatic castration-resistant prostate cancer: a prespecified subgroup analysis of the STRIVE trial
Source: Prostate Cancer Prostatic Dis. 2021 Oct 7;25(2):363–5. doi: 10.1038/s41391-021-00465-7 (PMC9184266; doi:10.1038/s41391-021-00465-7)
Supplement: Supplementary file 1 — Supplemental Materials [file 41391_2021_465_MOESM1_ESM.docx]

Supplementary Table S1. Patient demographics and baseline characteristics in the STRIVE nmCRPC subpopulation

| **Characteristic** | **Enzalutamide (*n* = 70)** | **Bicalutamide (*n* = 69)** |
| --- | --- | --- |
| Age, years |  |  |
| <65, *n* (%) | 11 (15.7) | 4 (5.8) |
| 65–74, *n* (%) | 25 (35.7) | 23 (33.3) |
| ≥75, *n* (%) | 34 (48.6) | 42 (60.9) |
| Mean (SD) | 73.1 (8.89) | 77.0 (7.46) |
| Median (range) | 73.5 (50.0–92.0) | 77.0 (58.0–91.0) |
| Race, *n* (%) |  |  |
| White | 53 (75.7) | 58 (84.1) |
| Black or African American | 15 (21.4) | 9 (13.0) |
| Asian | 0 | 1 (1.4) |
| Other | 2 (2.9) | 1 (1.4) |
| Baseline ECOG PS, *n* (%) |  |  |
| 0 | 56 (80.0) | 53 (76.8) |
| 1 | 14 (20.0) | 16 (23.2) |
| Baseline pain score by BPI-SF, *n* (%) |  |  |
| 0–1 | 59 (84.3) | 59 (85.5) |
| 2–3 | 11 (15.7) | 10 (14.5) |
| Disease stage at study entry per CRF, *n* (%) |  |  |
| M0/N0 | 61 (87.1) | 60 (87.0) |
| M0/N1 | 9 (12.9) | 9 (13.0) |
| PSADT, mo | *n* = 70 | *n* = 65 |
| Mean (SD) | 5.3 (4.18) | 7.9 (7.77) |
| Median (range) | 3.9 (0.6-23.6) | 5.3 (0.5-42.5) |
| PSADT category, *n* (%) | n = 70 | n = 65 |
| <3 mo | 23 (32.9) | 15 (21.7) |
| ≥3 to <6 mo | 25 (35.7) | 22 (31.9) |
| ≥6 mo | 22 (31.4) | 28 (40.6) |
| History of prior CV disease, *n* (%) |  |  |
| Yes | 22 (31.4) | 22 (31.9) |
| No | 48 (68.6) | 47 (68.1) |
| Baseline ECG result, *n* (%) |  |  |
| Normal | 21 (30.0) | 19 (27.5) |
| Abnormal, not clinically significant | 49 (70.0) | 50 (72.5) |

*BPI-SF* Brief Pain Inventory-Short Form; *CRF* case report form; *CV* cardiovascular; *ECG* electrocardiogram; *ECOG* *PS* Eastern Cooperative Oncology Group performance status; *nmCRPC* nonmetastatic castration-resistant prostate cancer; *mo* months; *PSADT* prostate-specific antigen doubling time; *SD* standard deviation.

Supplementary Table S2. Time to prostate-specific antigen progression in subgroups of the STRIVE nmCRPC population

|  | **Enzalutamide** | **Bicalutamide** | **Hazard ratio (95% CI)^a^** | ***P* value^b^** |
| --- | --- | --- | --- | --- |
| Overall PSA progression, median mo (% of patients) | NR (18.6)  *n* = 70 | 11.1 (65.2)  *n* = 69 | 0.18  (0.10–0.34) | <0.0001 |
| PSADT, <10 mo, median mo (% of patients) | NR (19.4)  *n* = 62 | 8.6 (76.0)  *n* = 50 | 0.16  (0.08–0.30) | <0.0001 |
| PDADT, ≥10 mo, median mo (% of patients) | NR (12.5)  *n* = 8 | NR (33.3)  *n* = 15 | 0.28  (0.03–2.44) | 0.221 |

Data cutoff date: 09 February 2015. *^a^*Hazard ratio was calculated using a unstratified Cox regression model with treatment as the covariate and is relative to bicalutamide with <1 favoring enzalutamide. ^b^*P* value was calculated using an unstratified log-rank test.

*CI* confidence interval; *NR* not reached; *mo* months; *PSA* prostate-specific antigen. *PSADT* PSA doubling time.

Supplementary Table S3. Prostate-specific antigen response rate (≥50% decrease from baseline) in subgroups of the STRIVE nmCRPC population

|  | **Enzalutamide** | **Bicalutamide** | **Difference (95% CI)^a^** | ***P* value^b^** |
| --- | --- | --- | --- | --- |
| Overall population, PSA response rate, %^c^ | 90.9% | 42.0% | 48.9%  (35.3%–62.4%) | <0.0001 |
| Patients with baseline PSA, no.  Evaluable patients^d^ | 70  66 | 69  69 | - | - |
| PSADT, <10 mo, % | 89.7% | 38.0% | 51.7%  (36.1%–67.2%) | <0.0001 |
| Patients with baseline PSA, no.  Evaluable patients^d^ | 62  58 | 50  50 | - | - |
| PSADT, ≥10 mo, % | 100% | 66.7% | 33.3%  (9.5–57.2%) | 0.071 |
| Patients with baseline PSA, no.  Evaluable patients^d^ | 8  8 | 15  15 | - | - |

^a^Difference calculated by enzalutamide rate minus bicalutamide rate. ^b^*P* values vs bicalutamide; comparison of the 2 treatment groups using an unstratified Cochran-Mantel-Haenszel mean score test.^c^Defined as ≥50% decrease from baseline. ^d^Evaluable patients for PSA response have a baseline PSA value and at least one postbaseline PSA value.
*CI* confidence interval; *mo* months; *nmCRPC* nonmetastatic castration-resistant prostate cancer; *PSA* prostate-specific antigen; *PSADT* prostate-specific antigen doubling time.

Supplementary Fig. S1. Subgroup analysis of progression-free survival in the STRIVE nmCRPC subpopulation


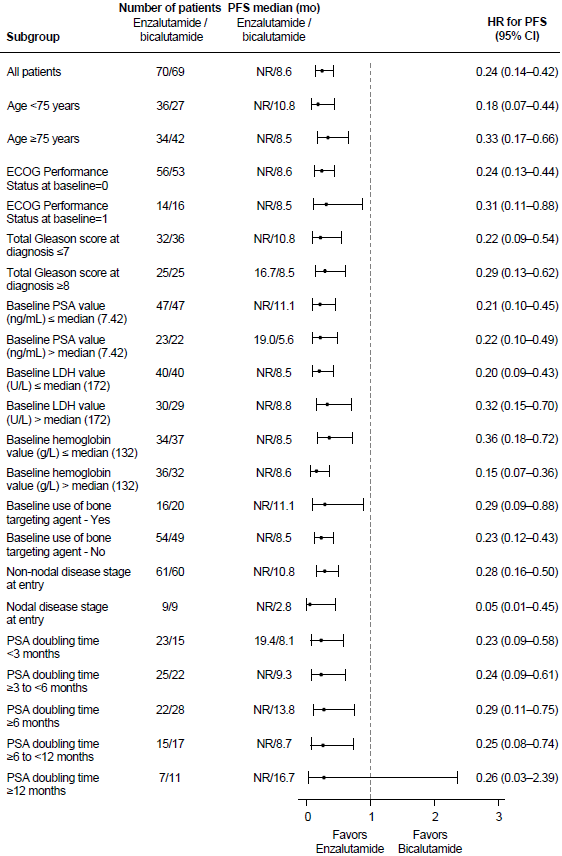


Hazard ratio was calculated using a unstratified Cox regression model with treatment as the covariate and is relative to bicalutamide with <1 favoring enzalutamide.

*CI* confidence interval; *ECOG* Eastern Cooperative Oncology Group; *HR* hazard ratio; *LDH* lactate dehydrogenase; nmCRPC nonmetastatic castration-resistant prostate cancer; *NR* not reached; *PFS* progression-free survival; *PSA* prostate-specific antigen.

Supplementary Fig. S2 Progression-free survival in the STRIVE nmCRPC population with PSA doubling times <10 months and ≥10 months.
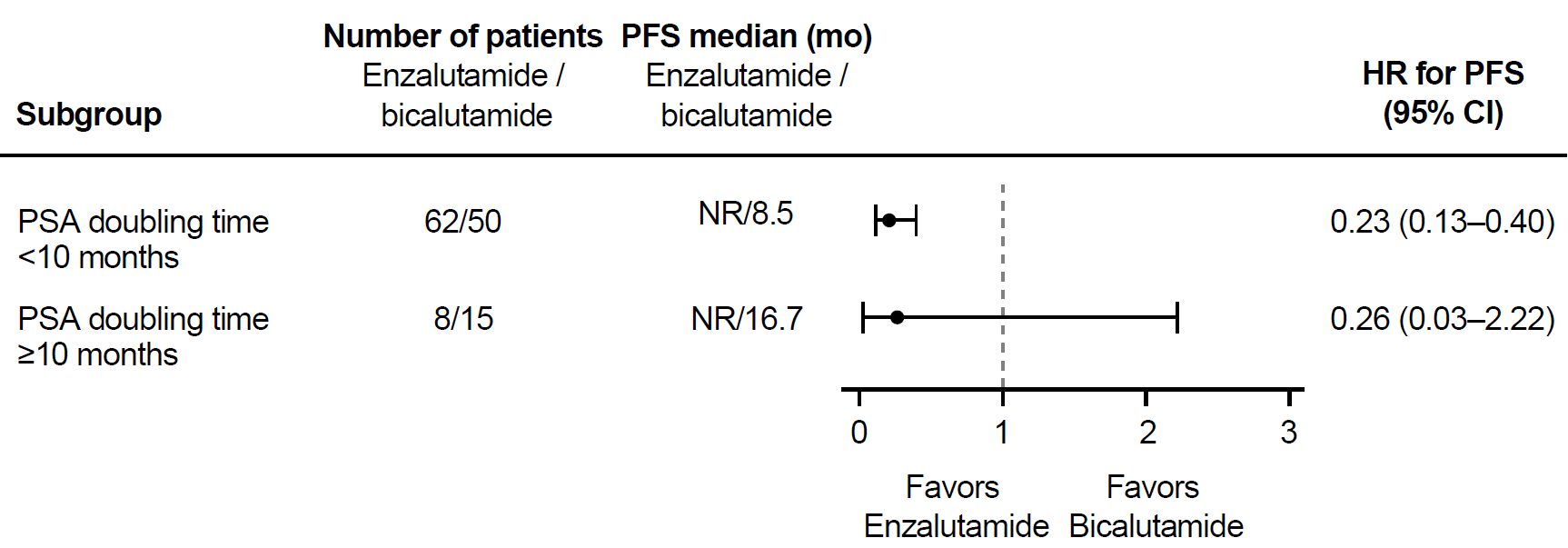


Hazard ratio was calculated using a unstratified Cox regression model with treatment as the covariate and is relative to bicalutamide with <1 favoring enzalutamide.

*CI* confidence interval; *HR* hazard ratio; *nmCRPC* nonmetastatic castration-resistant prostate cancer; *NR* not reached; *PFS* progression-free survival; *PSA* prostate-specific antigen.

Supplementary Fig. S3. Subgroup analysis of radiographic progression-free survival in the STRIVE nmCRPC subpopulation.


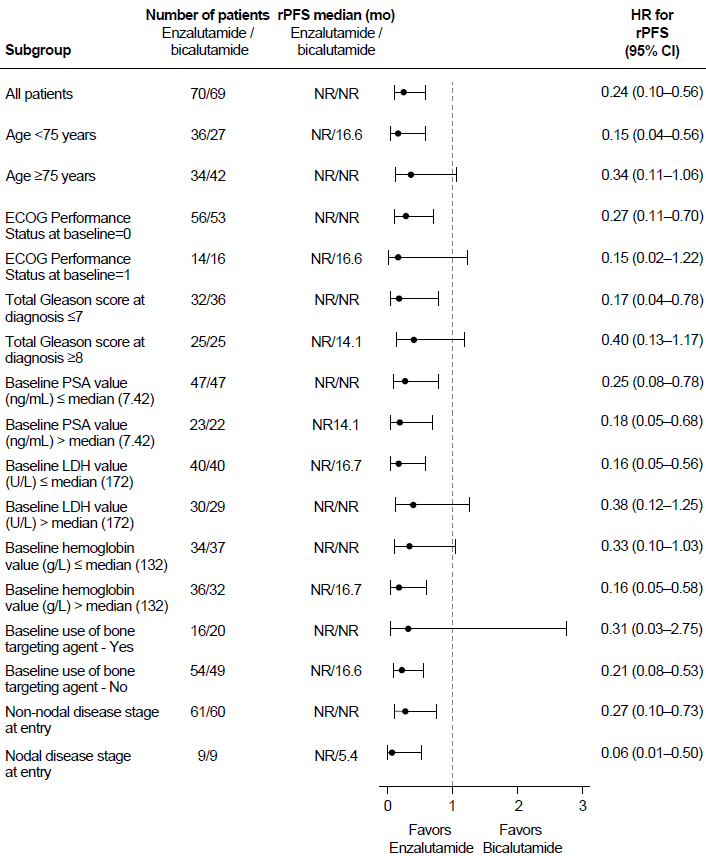


Hazard ratio was calculated using a unstratified Cox regression model with treatment as the covariate and is relative to bicalutamide with <1 favoring enzalutamide.

*CI* confidence interval; *ECOG* Eastern Cooperative Oncology Group; *HR* hazard ratio; *LDH* lactate dehydrogenase; nmCRPC nonmetastatic castration-resistant prostate cancer; *NR* not reached; *PSA* prostate-specific antigen; *rPFS* radiographic progression-free survival.
